# Failure patterns and survival in patients with nasopharyngeal carcinoma treated with intensity modulated radiation in Northwest China: A pilot study

**DOI:** 10.1186/1748-717X-7-2

**Published:** 2012-01-10

**Authors:** Jianhua Wang, Mei Shi, Yuesheng Hsia, Shanquan Luo, Lina Zhao, Man Xu, Feng Xiao, Xuehai Fu, Jianping Li, Bin Zhou, Xiaoli Long

**Affiliations:** 1 Department of Radiation Oncology, Xijing Hospital, Fourth Military Medical University, No.15 Changle West Road, Xi'an, China

**Keywords:** nasopharyngeal carcinoma, intensity modulated radiotherapy, chemotherapy, pattern of failure, survival

## Abstract

**Purpose:**

To evaluate the clinical outcomes and patterns of failure in patients with nasopharyngeal carcinoma (NPC) treated with intensity modulated radiotherapy (IMRT) in Northwest China.

**Methods and materials:**

From January 2006 to December 2009, 138 NPC patients were treated at Xijing Hospital. Of them, 25 cases with stage I-II received IMRT only, 113 cases with stage III-IVb received IMRT plus accomplished platinum-based chemotherapy. The IMRT prescribed dose was PTV 68-74 Gy to gross disease in nasopharynx and 66-72 Gy to positive lymph nodes in 30-33 fractions, and high risk and low risk region PTV was 60-63 Gy and 50.4~56 Gy in 30~33 and 28 fractions respectively. Plasma Epstein Barr virus (EBV) DNA load was measured before treatment. The clinical toxicities, outcomes and patterns of failure were observed.

**Results:**

The median follow up time was 23 months (range 2 to 53 months). EBV infection positive was only 15.9%. Overall disease failure developed in 36 patients, 99% belonged to stage III/IV disease. Among these, there were 26 distant metastases, 6 local recurrence, and 4 regional recurrence. The 3-year local control rate(LCR), distant metastasis-free survival (MFS), disease-free survival (DFS) and the overall survival (OS) was 93.9%, 79.5%, 70% and 83.1% respectively. Multivariate analyses revealed that age and anemia pre-radiotherapy were independent predictors for OS.

**Conclusion:**

IMRT with or without chemotherapy can improve the long term survival of NPC patients in Northwest China. Distant metastasis becomes the main cause of treatment failure. Age and anemia before radiotherapy were the main prognosis factors of NPC patients.

## Introduction

Nasopharyngeal carcinoma (NPC) presents a remarkably distinctive ethnic and geographic distribution, it is a rare malignancy in northwestern China but is more common in Southern China or Southeast Asia. The Epstein Barr virus (EBV) infection is frequently associated with the patients in Southern China [1,2]. NPC is highly sensitive to radiation, early stage NPC is often curable by radiation, and radiotherapy is the mainstay treatment strategy. However approximately 70% of newly diagnosed NPC patients present with stage III or IV disease, which are prone to suffer from locoregional recurrence or distant metastases after radiotherapy alone [3-5]. Hence, treatment of advanced NPC usually requires combined chemoradiation therapy. Forepart randomized trials displayed adjuvant chemotherapy delivered with conventional radiotherapy failed to improve NPC overall survival [6], those results were subsequently confirmed by a number of studies [7]. Although the neoadjuvant chemotherapy has demonstrated improved locoregional control and event-free survival, its benefit on overall survival has not yet to be confirmed [8]. In a word, it lacks powerful evidences to demonstrate overall survival benefits from chemotherapy [9], even through randomized trials of concurrent chemoradiation therapy for advanced NPC have showed a progression-free survival [5,10-12].

In pattern of conventional 2 dimensional radiotherapy, many factors are associated with the NPC prognosis, including age, gender, anemia, TNM stage, histopathology, radiation dose, radiation field, and combined manner of chemotherapy [1,13,14]. Nasopharynx is surrounded by more radiosensitive structures, simultaneity nasopharyngeal carcinoma cell is easy to infiltrate and spread towards surrounding normal organs. Those result in NPC irradiation target volumes are very irregular, conventional radiotherapy adopted lateral opposing fields for NPC rarely achieves the prerequisite dose and precision to tumor targets. On account of advancement of radicalized technology, especially IMRT has been applied, the limitation of conventional 2D radiotherapy for NPC can be overcome by IMRT, synchronously, traditional prognostic factors seem to change stealthily. IMRT can contribute to a high dose to the tumor while keeping down normal tissue complications by limiting radiation dose to normal tissues, the different organs can receive different fractional dose at the same fraction of treatment, results in the best radicalized biological effectiveness [15,16]. Several series reported showed excellent local control of more than 90% in NPC received with IMRT [16,17], even among patients with advanced T3-4 diseases [18]. Especially the phase II 0225 Trial confirmed the excellent outcomes, IMRT with or without chemotherapy in the treatment of NPC achieved the almost indistinctive benefit in local progression-free and overall survivals [19].

Even though numerous favourable reports for NPC have focus on IMRT, IMRT can unfold a novel threshold for NPC, the result still need to be confirmed in different region. The present retrospective study exhibited clinical outcomes and patterns of failure in NPC patients treated with IMRT in Northwest China.

## Materials and methods

All patients were native and resided in northwest China, consisted of 138 consecutive untreated patients who had histologically confirmed NPC before radiotherapy between January 2006 and December 2009. Stage I-IVb NPC histologically classified by the World Health Organisation (WHO) system [20]: Stage I, representing squamous cell carcinoma, is similar to carcinomas arising from other sites of head and neck. Stage II is characterised as non-keratinizing carcinoma. Stage III, representing undifferentiated carcinoma. TNM classification system by AJCC [21,22]. Patients who had evidence of distant metastasis were remove from this treatment choice. Of those patients. 25 cases with stage I-II received IMRT only, 113 cases with stage III-IVb received IMRT plus accomplished platinum-based chemotherapy. Patient characteristics and treatment factors are shown in Table 1.

**Table 1:** Patient and disease characteristics

	Patients	
	
Characteristics	**No**.	%
Age, years		
Median	46	
Range	11-72	
Sex		
Male	104	75.4
Female	34	24.6
EBV infection		
< 1000 copy	116	84.1
≥1000 copy	22	15.9
Tumor factors		
WHO histology		
I	2	1.5
II	99	71.7
III	37	26.8
AJCC T category		
T1-2	100	72.5
T3-4	38	27.5
AJCC N category		
N0-1	78	56.5
N2-3	60	43.5
AJCC stage		
I	6	4.4
II	19	13.8
III	64	46.4
IVa	39	28.3
IVb	10	7.3
Treatment factors		
IMRT alone	25	18.1
IMRT + chemotherapy	113	81.9
Chemotherapy manner		
Neoadjuvant chemotherapy	43	31.2
Concurrent chemotherapy	18	13.0
Concurrent +Adjuvant	52	37.7

WHO = World Health Organization.

AJCC = American joint committee on carcinoma

Concurrent +Adjuvant = Concurrent +Adjuvant chemotherapy

Pretreatment evaluations consisted of complete physical examination, nasopharyngoscopy, Magnetic resonance imaging (MRI) or computed tomography (CT) imaging of the nasopharyngeal region and neck. Positron emission tomography (PET) was optional. The routine examine included chest X-ray, complete blood count, serology of Epstein-Barr virus (EBV), renal and liver function tests. Anaemia assumed the HBC value: < 120 g/L in males, < 110 g/L in females. Additional investigations were performed for those with suspicious findings or abnormal biochemical profile.

## Methods

### IMRT

All patients were immobilized in the supine position with thermoplastic masks. Contrast-enhanced planning computed tomography (CT) scans with a 3 mm slide thickness were then obtained, with coverage from the skull vertex to 2 cm below the clavicles. MRI/CT images was performed for all patients for accurate delineation of tumor volumes and critical structures. The primary tumor and upper neck above the bottom of hyoid bone was treated with IMRT techniques using seven coplanar beams, inverse treatment planning was performed using the Eclipse treatment-planning system with simulated annealing. IMRT was delivered by using a simultaneous-integrated boost (SIB) technique [23].

The gross tumor volume (GTV) includes the primary nasopharyneal tumor (GTVnx) and involved lymph nodes (GTVnd) as demonstrated by imaging and physical examinations. The high-risk clinical tumor volume (CTV-1) included GTV plus 5 mm margin and encompassed the entire nasopharyngeal mucosa plus 5 mm submucosal volume. The CTV2 covers the CTV1 and area at risk, including posterior third of nasal cavity and maxillary sinus, pterygopalatine fossa, posterior ethmoid sinus, parapharyngeal space, skull base, clivus or based on tumor invasion. The CTV3 covers lower risk lymphatic levels. The planning target volume (PTV) was created based on each CTVs with an additional 3-5 mm margin, accounting for organ motion/daily treatment set-up uncertainties. In areas where the GTV or the CTV was adjacent to critical normal structures (ie, brainstem), a smaller margin was delineated.

The prescribed dose was 68-74 Gy to gross disease PTV in nasopharynx and 66-72 Gy to positive lymph nodes in 30-33 fractions, and the prescribed dose to high risk vs. low risk region PTV was 60-63 Gy vs. 50.4~56 Gy in 30~33 and 28 fractions respectively. Treatment will be delivered once daily, 5 fractions per week. The dose received by each OAR should be no more than its tolerance limits. For patients given induction chemotherapy, the target delineations were based on the pre-chemotherapy extent as shown on the CT/MRI images.

### Chemotherapy

Except 25 patients in stage I-II, other 113 patients in stage III-IVb had additional accomplished platinum-based chemotherapy (Table 1). Various sequences and regimens of cytotoxic drugs (all cisplatin based) had been used. Neoadjuvant chemotherapy was given to 43 patients, who were in stage III~IVb, mainly consisted of 2~3 cycles of PF (cisplatin 30 mg/m2/d IV for 3 days, 5-FU 800~1000 mg/m2 IV in d1-d5) or TP regimen (Docetaxel 75 mg/m2 IV in d1, cisplatin 30 mg/m2/d IV for 3 days) at a 2 week interval prior to the initiation of RT treatment. Concurrent chemotherapy only was given to 18 patient mainly with cisplatin 80-100 mg/m2 on day 1-3 at 3 weekly intervals during the course of RT. Concurrent and adjuvant chemotherapy were given to 52 patients according to NPC-9901 Trial. Patients who received neoadjuvant chemotherapy were not offered adjuvant chemotherapy.

### Follow-up

All patients were evaluated weekly during treatment period, after the completion of their treatment, followed-up every 3 months in the first 2 years, every 6 months between 2-5 years. Each follow-up included a complete examination, basic serum detection, chest X-ray, and ultrasound of liver and abdomen. Endoscopy was performed at every visit after treatment. MRI of the head and neck areas was performed every 6 months. PET was optional in high risk incident. Toxicities were observed and scored according to the Toxicity criteria of the Radiation Therapy Oncology Group (RTOG) radiation morbidity scoring criteria at each follow-up [24].

The primary endpoints were treatment compliance and acute toxicities. The secondary endpoints of this study were late toxicities, local recurrence-free survival (LRF), regional recurrence-free survival (RRF), distant metastasis-free survival (DMF), disease-free survival (DFS) and overall survival (OS). Follow-up time was ensured from the date of treatment initiation to the date of the last contact or death. Time to failure was calculated from the date of treatment initiation to the date of the relevant event. Survival analyses were computed using the Kaplan-Meier method, P < 0.05 was considered significant. The analyses were performed with the SPSS software package (Version 13.0, SPSS Inc., Chicago, IL).

## Results

### Patterns of failure

The total numbers of death were 17 cases, including tumor recurrence (3 patients), distant metastasis (9 patients), both recurrence and metastasis (5 patients).

**Figure 1:**
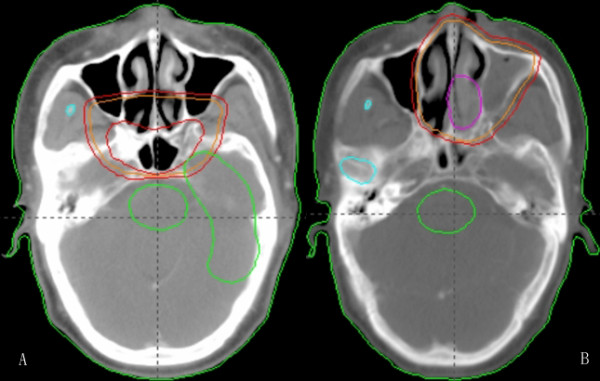
**Intensity modulated radiotherapy plan for nasopharyngeal carcinoma**. A: Before treatment; B: Failures were marginal-field after treatment.

Table 2 lists the patterns of failure. The overall failure rate was 26.1% (36 patients): among these, 6 local recurrence, 4 regional recurrence, noticeable event was only 2 cases local/regional recurrence were marginal of field radiation (Figure 1), 8 patients had inside-field recurrence. The median recurrent time was 16 months (range, 4-23 months). There were 26 distant metastases (18.8%), the most common metastasis site is bone. The median distant metastasis time was 9.5 months (range, 3-23 months). Obviously, distant metastasis was the major patterns of failure.

**Table 2:** Causes of failure

No. of patients Site	n	%
Relapse	10	
local relapse	6	4.3
Regional relapse	4	2.9
Metastasis	26	
Bone	12	8.9
Liver	2	1.5
Lung	6	4.4
Other location	2	1.5
Multiple location	4	2.9

### Toxicities

The IMRT plans showed decreased doses to normal structures and increased doses to target volumes, which might decreased the unwanted side effects. The acute toxicities were listed in Table 3. The primary toxicities were xerostomia, stomatitis, dermatitis and neutropenia, which were generally mild or moderate. The total incidence of grade 3 acute toxicities in patients received IMRT plus chemotherapy was 22 patients (19.5%), significantly higher than those received IMRT alone 2 patients (8,0%) (P < 0.05).

**Table 3:** Acute toxicities during treatment

Acute toxicities	I (%)	II (%)	III(%)
Dermatitis	29(21.0)	20(14.5)	7(5.1)
Stomatitis	16(11.6)	8(5.8)	3(2.2)
Neutropenia	24(17.4)	13(9.4)	2(1.5)
xerostomia	28(21.1)	26(18.8)	12(8.7)

The late toxicities include Grade II mucositis 3 patients (2.2%), Grade III mucositis 14 patients(10.1%), Grade III xerostomia 16 patients (11.6%), Grade III leucocytopenia 6 patients(4.3%).

No grade IV acute and late toxicities were detected.

### Survival analysis

The median follow-up time was 23 months (range, 2-53 months). 3-year OS, LRF, RFS, MFS and DFS were 83.1%, 93.9%, 96.3%, 79.5% and 70%, respectively. Locoregional recurrences did tend to occur more frequently in the first 2 years post-treatment, resulted in the 2-year OS fall rapidly.

**Figure 2:**
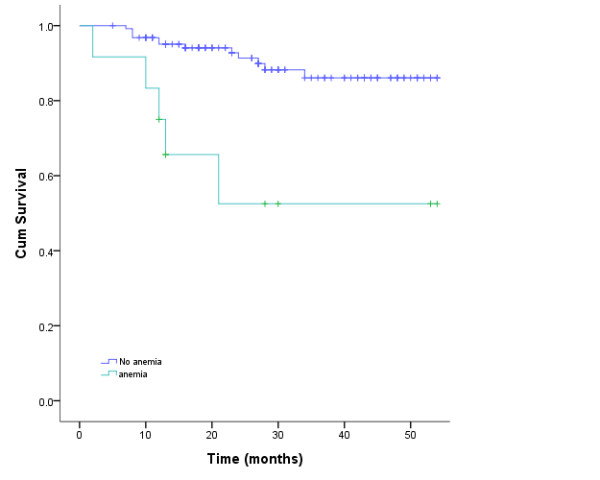
**Kaplan-Meier estimate of anemia factorial survival for nasopharyngeal carcinoma patients treated with intensity modulated radiotherapy**.

**Figure 3:**
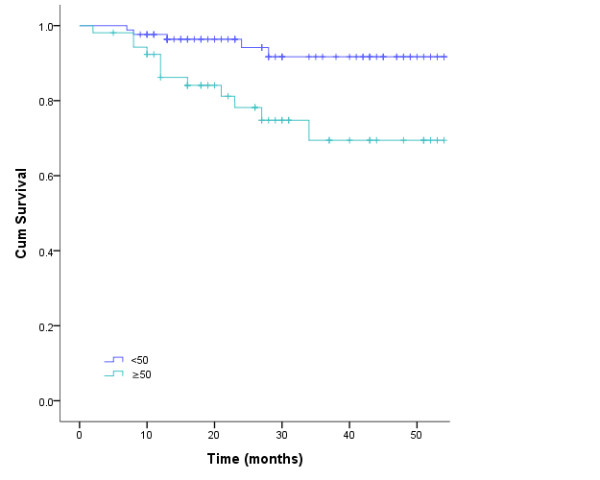
**Kaplan-Meier estimate of age factorial survival for nasopharyngeal carcinoma patients treated with intensity modulated radiotherapy**.

The value of various potential prognostic factors include age, gender, stage and use of chemotherapy on predicting local control, DMF, DFS, and OS rates were evaluated. Univariate analysis of prognostic factors in survival rates showed male, stage T or N, lymph node biopsy and chemotherapy was not associated with local control, DFS, MFS and OS; Anemia before radiotherapy and age (≤ 50 vs. > 50) were found to be the independent predictors for OS (P = 0.013, hazard ratio [HR] 4.33, 95% confidence interval [CI] 1.367~13.734; P = 0.001, hazard ratio [HR] 6.99, 95% confidence interval [CI] 2.290~21.343). To adjust for the prognostic factors, the Cox regression analysis confirmed the outcomes (Figure 2, 3).

IMRT with or without chemotherapy provided no significant additive effect on local/regional control, 3-year MFS, DFS, and OS rates in this group of patients with advanced NPC, regardless of their T-classification or nodal status, definitively treated with IMRT in multivariate analysis (P > 0.05). Distant metastasis becomes the main cause of treatment failure.

## Discussion

NPC presents high sensitivity to radiation. Radiation alone for early NPC can achieve relatively high cure rate, however, it is disappointing for locally advanced disease. In pattern of conventional 2 dimensional radiotherapy, increasing evidences support concurrent chemoradiation to be standard treatment strategy for locally advanced NPC, its efficacy has been proven in a number of randomized phase III trails and meta-analyses [12,13,25]. However, it's difficult to increase OS by these arms.

IMRT has been demonstrated often to produce superior dose distributions in terms of improved tumor coverage and lower doses to normal tissues for a variety of cancers originating in the NPC region. IMRT allows for fine modulation of radiation intensity within each radiation beam, which can achieve high dose in tumor without overdosing the normal organs [16,17]. It has been proven that IMRT techniques is superior to conventional technique with respect to technical advantages, local or regional control, critical organ sparing and treatment outcomes [26]. IMRT has been proven excellent in the NPC treatment, patients with at least 66.5 Gy to their target volumes had significantly less locoregional failure. The last randomized study showed that IMRT with different chemotherapy sequence produced similar outcomes in terms of OS, DFS, RFS and MFS rates [19]. Similarly, thus excellent results and less toxicities in our patients could be largely due to the IMRT applied. However, an unimagined univariate analysis showed that IMRT eliminated many independent prognostic factors compared with conventional radiotherapy, only age > 50 years and anemia before radiotherapy were significantly associated with poor OS; Male, stage T or N and lymph node biopsy was not associated with RFS, DFS, MFS and OS. Multivariate analysis of prognostic factors in survival rates showed the same results, which implied IMRT did balance the effects of different chemotherapy pattern. The results maybe benefit from high dose distributing for tumor, and significant improvement profited from the IMRT. Especially, chemotherapy failed the independent prognostic factor, which presented more different from many randomized trials [1,13,14,27,28]. The present study implied concurrent and adjuvant chemotherapy provided no significant additive effect on local/regional control, MFS, DFS, and OS rates in advanced NPC patients, regardless of their T-classification or nodal status, definitively treated with IMRT. A logical interpretability did bring IMRT into prominence; Secondly likelihood attributed to small sample size or nonrandom incident in present study; Other perhaps tumor characteristic in Northwest China represent the favorable prognosis compared with the disease in Southeast China [26,29]. EBV and histological types in Northwest are significant different from those in Southeast China. Patients present high EBV infection > 90% and major undifferentiated tumor in Southeast China, and EBV is a well-established risk factor for NPC [29] particularly in undifferentiated NPC [30]. Conversely, the present study showed that the EBV infection was only 15.9%, the undifferentiated NPC was only 26.8% and all patients with EBV-positive were undifferentiated NPC, which implied EBV play an important role in the aetiology of this NPC histological type. Perhaps it caused the diversity prognosis of NPC between Southeast and Northwest China. High EBV prevalence in undifferentiated NPC prompts EBV should be a risk and poor prognosis factor for NPC [31,32]. Even so, two cases local/regional failures in the study were still marginal of field radiation, and failed to rescue by chemotherapy, which prompted the marginal of field should be considered carefully to broaden befittingly in high-risk patients.

The present data investigated, for the first time in Northwest China, the role of EBV in the aetiology of NPC has been eliminated. The findings prompted NPC in the low-incidence areas rarely represented undifferentiated carcinoma, which maybe include other nosogenesis, are different from Southeast China.

In summary, the present study showed NPC in Northwest China possesses remarkable different from NPC in Northwest, primary distinction represent in aetiology, pathology and prognosis. IMRT in Northwest China has achieved excellent results as well as an acceptable acute toxicity profile.

## Competing interests

The authors declare that they have no competing interests.

## Authors' contributions

SL, XF, FX, JL, XL, BZ and MX participated in the treatment panning, contributed to the data collection. MS participated in its design and coordination and helped to draft the manuscript. JW, RL and YH conceived of the study, participated in the treatment panning, performed the statistical analysis, and drafted the manuscript. All authors read and approved the final manuscript.
